# Developing a codebook for assessing auditory hallucination complexity using mixed methods

**DOI:** 10.3389/fpsyt.2024.1441919

**Published:** 2024-12-12

**Authors:** Igor J. Pietkiewicz, Radosław Tomalski, Anna M. Hełka

**Affiliations:** ^1^ Research Centre for Trauma and Dissociation, Ignatianum University in Cracow, Cracow, Poland; ^2^ Insititute of Psychology, SWPS University, Warsaw, Poland

**Keywords:** concept mapping, mixed methods, interview for voice-hearers, assessment, voice complexity, auditory hallucinations

## Abstract

**Introduction:**

In recent years there has been a notable expansion of psychotherapeutic approaches to treat people experiencing auditory verbal hallucinations (AVH). While many psychotherapists conceptualize voices as “dissociative parts” and apply therapeutic techniques derived from the field of dissociation, research investigating AVH from this perspective is limited. Despite the acknowledgment that voices encountered in dissociative identity disorder (DID) often exhibit high complexity and autonomy, there is a critical need for assessment tools capable of exploring voice complexity across different clinical groups. Such tools hold significant potential for aiding clinicians to identify patients who may benefit more from dissociation-based therapy approaches. This study aims to operationalize the concept of voice complexity (VC) by identifying its different dimensions and indicators.

**Methods:**

Using concept mapping procedures, 12 healthcare professionals and two voice-hearers participated in brainstorming, and 24 people with clinical backgrounds performed sorting and rating tasks.

**Results:**

Seven dimensions of VC were identified: System Complexity, Content Complexity, Voice’s Interest Complexity, Interaction Complexity with Voice-Hearer, Voice’s Own Life, Voice Influence, and Voice’s Vocal Characteristics. A codebook for assessing VC with indicators for varying levels of complexity across these dimensions was developed and can be used with the Structured Clinical Interview for Voice-Hearers. Inter-rater reliability, measured by comparing the assessments of two interview transcripts by seven raters using Kendall’s Coefficient, indicated substantial agreement in one interview (W = .613) and almost perfect agreement in the second (W = .805).

**Discussion:**

The new instrument has promise as an effective tool for comparative studies exploring VC in diverse clinical and non-clinical populations, with potential implications for clinical practice and future research.

## Introduction

Auditory verbal hallucinations (AVH) represent a complex and multifaceted phenomenon within clinical psychology and psychiatry, extending beyond their traditional association with psychosis. They are increasingly recognized across a spectrum of psychiatric disorders, spanning dissociative disorders, personality disorders, post-traumatic stress disorder, eating disorders, substance abuse, and even among non-clinical populations (1). While some authors argue for similarities in voices across different diagnostic groups (2), recent studies suggest a more nuanced understanding of this phenomenon (3).

AVH appear to arise from various psychological and neurobiological mechanisms, resulting in significant heterogeneity. This diversity suggests the potential need for more tailored or individualized psychotherapy approaches for individuals experiencing auditory hallucinations who seek treatment. One concept that may be particularly useful in adapting psychotherapeutic approaches is that concerning the complexity of voices.

Therapists working with individuals who experience auditory verbal hallucinations (AVH) often conceptualize these phenomena as “dissociative parts” and apply psychotherapeutic techniques borrowed from the field of dissociation (4). However, there is currently a lack of clear therapeutic guidelines regarding when this approach should be employed and when it might prove ineffective. The concept of a “dissociative part” originates from the theory of structural dissociation of personality (5), which builds upon Pierre Janet’s theory. Dissociative parts, as observed in dissociative identity disorder (DID), are characterized by a high degree of mental autonomy and elaboration, encompassing a sense of separate self, skills, memory, and other features. While dissociative parts can manifest as voices, they may also produce various other symptoms in complex dissociation, such as passive influence phenomena (intrusions) or memory gaps. Despite the relevance of dissociative parts to AVH, there have been limited attempts in AVH research to link this concept to the diverse experiences of hearing voices. Recent developments in this direction include the identification of new descriptive categories, such as characterful voices (6), and the level of personification, distinguishing between minimal and complex personification (7). Complex personification involves multiple person-like qualities, including elaborate descriptions of intentional states, agency, and identity. This complexity is not solely determined by the frequency, quantity, or topic of speech but is based on phenomenological features and the voice-hearer’s subjective perspective. However, while these distinctions offer valuable insights, they lack a clear theoretical foundation and may have limited clinical utility due to a voice-hearer’s or researcher’s subjective interpretation. In contrast, anchoring the concept of voice complexity in the theory of the structural dissociation of personality (5) could offer a theoretically grounded framework. This approach has the potential to reflect the extent of dissociative mechanisms, particularly compartmentalization (8), in the experience of hearing voices. Thus, further development of the concept of voice complexity may prove useful for treatment planning, and personalizing psychotherapeutic strategies for individuals who hear voices.

To delve into VC, it is essential to gather in-depth narratives of voice-hearing experiences alongside concurrent dissociative symptomatology. This is best achieved through the use of semi-structured clinical interviews (9). In this context, our study aims to employ mixed methods to elucidate the concept of voice complexity, delineate its dimensions and indicators, and develop and test a codebook suitable for the assessment of auditory verbal hallucinations (AVH).

## Methods

This study aims to investigate the multifaceted nature of voice complexity, identifying its domains and indicators, and to develop a comprehensive codebook for evaluating the complexity of auditory hallucinations. Following the approval of the university ethics board, we employed group concept mapping (10), an integrated participatory mixed-methods approach that involved actively engaging clinicians and voice hearers as co-researchers in the design, analysis, and interpretation of the research. This ensured that their experiences and insights directly informed the study’s outcomes. We conducted a series of structured steps, encompassing preparation, brainstorming, sorting and rating, representation, data analysis and interpretation, and utilization.

The participants engaged with transcripts of the Structured Clinical Interview for Voice-hearers (SCIV, 11) delving into the experiences of auditory hallucinations. They then used an online platform to generate statements they deemed indicative of low, medium, or high voice complexity. A refined compilation of these statements formed the basis for subsequent sorting and rating tasks performed during a group workshop, culminating in the development of a concept map — a visual representation delineating theoretical clusters pertaining to voice complexity. Subsequently, seven raters independently assessed the two SCIV transcripts using the developed codebook. Their responses were then compared to evaluate inter-rater reliability, with statistical analyses enhancing the robustness of this study by providing empirical support for the consistency of the raters’ assessments.

### Concept mapping procedures

#### Preparation

This step entailed familiarizing the participants with the concept of voice complexity, achieved through the presentation of interview transcripts from two individuals: one diagnosed with schizophrenia and another with dissociative identity disorder. This complexity manifested through various phenomenological features, including the voices’ characteristics, content, or the relationship with the voice-hearer. A prompt was formulated to solicit their insights regarding various phenomenological features of auditory hallucinations, aimed at discerning indicators for low, medium, or high levels of complexity: “What might serve as indicators for low, medium, or high voice complexity?”

#### Recruitment for brainstorming

Invitations were extended to 42 healthcare professionals who had previously undergone specialized training in clinical assessment of trauma-related conditions or in psychotherapy for psychosis, in collaboration with the Research Centre for Trauma & Dissociation. These professionals regularly supervised clinical work and possessed experience in treating patients with auditory hallucinations. Twelve professionals responded and participated in the brainstorming activity. In line with the participatory research ethos, invitations were also extended to individuals with a history of psychiatric treatment who experienced auditory hallucinations, were characterized by being well-functioning, articulate, and open to engage in participatory research. Two of them agreed to participate in brainstorming.

During the brainstorming task, data were collected via a dedicated online application www.e-psyche.eu where participants completed a demographic questionnaire. The questionnaire included questions about their age, gender, professional background and experience, whether they knew people hearing voices, or heard voices themselves. Among the 12 healthcare professionals, there were ten women and two men, ages between 29 and 50 (M = 41.83, SD = 5.18). All of them were Caucasian, had university degrees (nine in psychology, three in medicine). They had between three and 25 years of clinical experience (M = 13.17, SD = 4.44). Two of them declared hearing voices. The two non-professionals who were voice-hearers were Caucasian men (ages 26 and 33), one had a university degree in sociology and the other in psychology.

#### Brainstorming

In this task, participants were encouraged to familiarize themselves with the two SCIV transcripts mentioned earlier. Healthcare professionals were also prompted to reflect upon their own patients who experience auditory hallucinations, while voice-hearers were encouraged to draw upon their personal experiences and compare them with those described in the transcripts. Subsequently, participants were directed to use the online platform to record statements that addressed the prompt (see Preparation). Each participant completed this brainstorming activity individually, resulting in the generation of a list of 173 statements. Participants were unable to view the responses of other participants. Upon reaching content saturation, where no new ideas pertaining to levels of voice complexity emerged, the brainstorming activity was closed.

#### Recruitment for sorting and rating

Only healthcare professionals were invited to undertake the sorting and rating tasks during a clinical workshop. Out of 48 who were sent invitations, 28 registered and completed both tasks, but four individuals submitted incomplete response sheets which were excluded from the analysis. They completed the same demographic questionnaire which was used earlier. The group consisted of 18 women and six men, with ages ranging from 26 to 58 years (M = 44.75, SD = 3.20), who were engaged in clinical practice from two to 25 years (M = 12.54, SD = 3.85) and had patients with auditory hallucinations. One of them self-disclosed hearing voices.

#### Sorting and rating

In this task, the authors meticulously reviewed every statement, eliminating duplicates or irrelevant ones (those unassociated with the prompt). They conducted preliminary sorting by categorizing statements into thematic groups, consolidating some items, and ensuring that each category contained statements describing various levels of complexity (from very low to very high). Following a review of the statements by other experts in the field, a master list of 32 items was created for the subsequent sorting and rating tasks. The aim of the sorting task was to verify if items relating to similar aspects of the voice-hearing experience would be grouped by participants into the same thematic categories and how participants would define these dimensions of voice complexity. During a clinical workshop, each participant received statements on separate pieces of paper, was requested to review and sort conceptually similar statements into piles and label each pile. After completing the sorting task, each participant was tasked with organizing statements within each pile based on their level of complexity, arranging them from the statement indicating the lowest complexity at the bottom to the most complex one at the top. They were then instructed to rate each statement on a 5-point Likert scale, answering the question: “What level of voice complexity does this feature indicate?” (1 = very low; 5 = very high).

### Analyses

#### Representation

A visual concept map was constructed to illustrate the final theoretical model of dimensions of voice complexity, based on how participants categorized brainstormed statements during the sorting task. Each participant created a matrix in which a “1” was coded in cells for statements sorted into the same pile. These matrices were combined across all participants, resulting in a 123 × 123 matrix where larger cell counts indicated statements sorted together more frequently. Using non-metric multidimensional scaling, each statement was assigned coordinates (x,y) in a two-dimensional space using an algorithm within the CM program. This algorithm aimed to place statements sorted together more often closer to one another and statements rarely or never sorted together farther apart. The resulting point map, based on the Kruskal and Wish (12) method, provides a two-dimensional representation of the sorting data. The stress value, which indicates how well the multidimensional scaling analysis fits the data, was 0.28, indicating a good fit and congruence between the processed and raw data (13).

#### Data analysis and interpretation

A hierarchical cluster analysis (14) was employed to empirically identify clusters of statements using CM software, based on their two-dimensional coordinates. This process confirmed our initial conceptualization. Most participants clustered statements into similar groups, suggesting that the indicators for VC effectively described a specific theme. However, two items did not align identically across all participants, prompting the research team to consider whether they should be reformulated. Nonetheless, we retained them in their original form within clusters defined by most participants.

The authors then revisited names which participants proposed for each cluster based on the content of statements comprising them. Similar to how closely located points on the map indicated statements sharing similar content, clusters depicted on the final model map also represented analogous content. We scrutinized the clusters within the final model, deliberating on potential relationships among clusters. Subsequently, we undertook an additional analysis, using participants’ ratings of statements.

While other types of analyses, such as those based on participants’ demographic data or roles, or involving additional questions to rate statements, are traditionally performed in CM (10), we deemed such analyses unsuitable for developing a codebook in our study. The primary rationale for employing CM in this process was the robustness of group brainstorming, allowing us to ascertain which aspects of voice complexity particular statements represent according to participants, and to assess their agreement on whether specific statements indicate high or low voice complexity.

## Results

**Figure 1:**
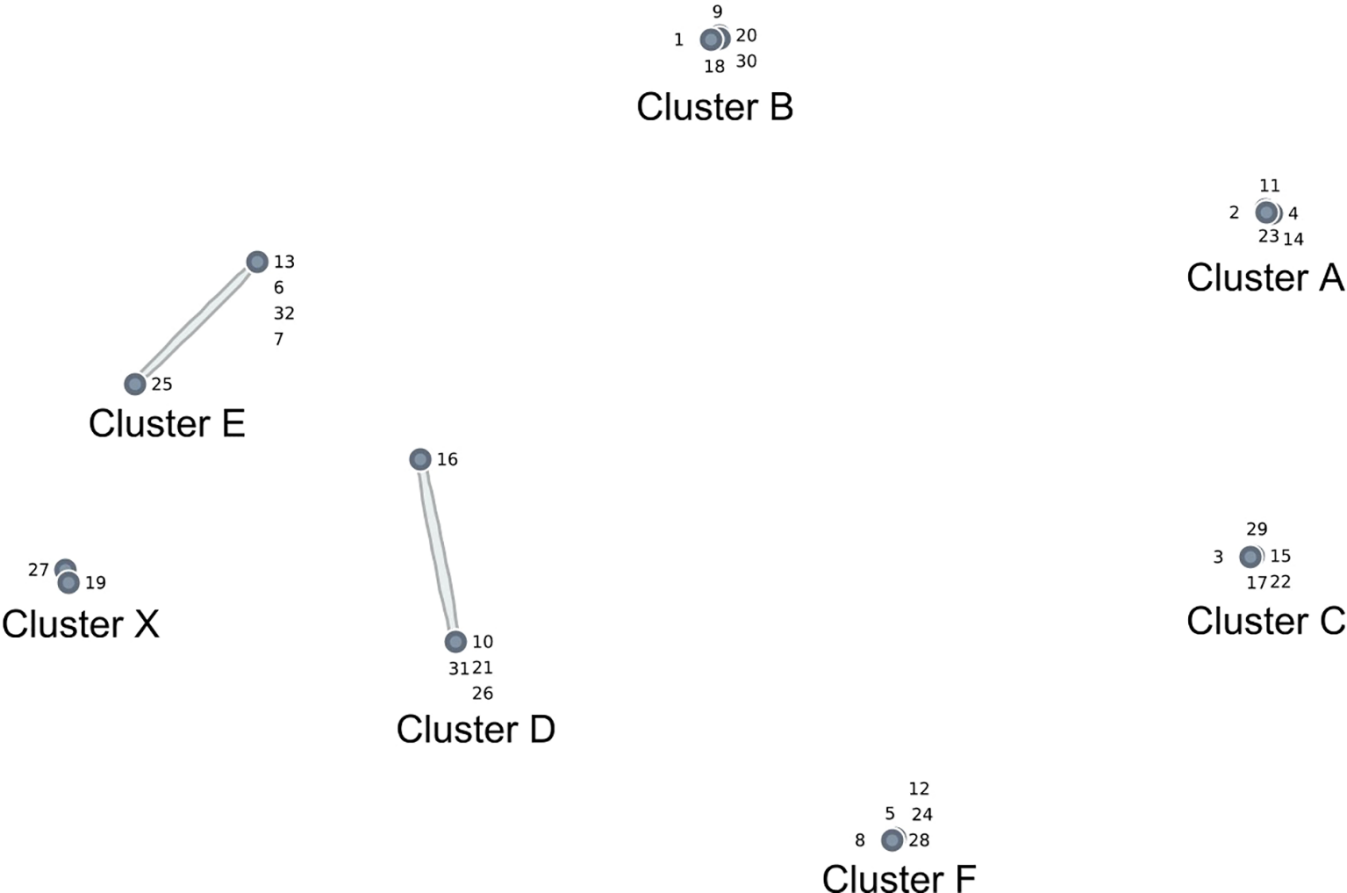
Voice complexity cluster map.

The final cluster map (see **Figure 1**) illustrates seven clusters of statements, each representing distinct dimensions of voice complexity. All clusters comprised five statements, except for Cluster X, which contained only two. Below is a summary of the clusters and statements, along with their respective ratings. The statements have been ordered based on their rating values, reflecting the level of complexity, ranging from very low to very high.

### Cluster A: system complexity

Statements in this cluster referred to the complexity of the internal system of the voice-hearer, expressed by the number of voices and the intricacy of interactions between them (**Table 1**).

**Table 1:** Cluster A: system complexity.

ID #	Statement	Mean score
23	A person experiences one voice.	1.3333
4	One experiences several different voices which do not interact or know about each other’s existence.	2.4167
11	One experiences several different voices with simple and repetitive patterns of interaction (e.g., attacking each other).	3.1667
14	One experiences several different voices, with complex interactions, and their patterns vary depending on the situation (e.g., sometimes they attack each other and other times they are in agreement).	4.0417
2	There are a dozen or dozens of voices which can be organized into different groups or subsystems. There are complex interactions between them which change over time.	4.9583

### Cluster B: content complexity

This cluster refers to the content of auditory hallucinations, spanning from basic, content-less sounds to sophisticated statements capable of dynamic evolution (**Table 2**).

**Table 2:** Cluster B: content complexity.

ID #	Statement	Mean score
20	No verbal content, simple sounds (e.g. noise, rustling, squealing, crying, screaming, knocking, music).	1.0417
30	Verbal content is difficult to identify, containing incomprehensible statements (e.g. whispers, chatter).	1.8750
9	Poor content limited to simple words or short, repetitive phrases.	2.6667
1	The content of voices includes complex statements, comments, or opinions on limited topics.	3.7917
18	The content of voices includes complex statements, comments, or opinions on various topics that change dynamically during the conversation.	4.7500

### Cluster C: voice’s interest complexity

This cluster relates to the interests of voices, spanning from scenarios where the voice-hearer cannot discern the focus of the voices to highly elaborate experiences where the voices’ attention is directed towards the voice-hearer, their experiences, or other voices (**Table 3**).

**Table 3:** Cluster C: voice’s interest complexity.

ID #	Statement	Mean score
3	It is not possible to determine voices’ object of interest.	1.2917
17	The voice focuses on the voice-hearer and regulating his or her behavior or emotions.	2.4167
29	The voice focuses on the voice-hearer, other people, or situations.	2.8333
15	The voice focuses on the voice-hearer, other people or situations, and its own emotional states, needs, or plans.	4.0417
22	The voice focuses on the voice-hearer, other people, or situations, its own emotional states, needs or plans, and the experiences of other voices.	4.8333

### Cluster D: interaction complexity with voice-hearer

This cluster relates to the interests of voices, spanning from scenarios where the voice-hearer cannot discern the voices’ focus to highly elaborate experiences where the voices’ attention is directed towards the voice-hearer, their experiences, or other voices (**Table 4**).

**Table 4:** Cluster D: interaction complexity with voice-hearer.

ID #	Statement	Mean score
16	The voice does not respond to the voice-hearer (e.g. his or her attempts to establish contact) or no such attempts have been made.	1.4167
31	The voice understands the questions or content addressed to it and responds in a simple way (e.g., gives casual answers, makes faces or gestures, or changes behavior).	2.3750
10	The voice understands the questions or content addressed to it and one can start a simple conversation with it (a sequence of several two-way statements).	2.9130
21	The voice understands the questions or content addressed to it and it is possible to maintain a fluent conversation with it for a longer period.	3.8333
26	The voice understands the questions or content addressed to it and it is possible to maintain a fluent conversation with it for an extended period, and physical contact is possible (e.g. touching, stroking, hugging, hitting the voice).	4.8333

### Cluster E: voice’s own life

This dimension concerns the degree of mental autonomy exhibited by voices, ranging from a lack of individual identity, thoughts, or emotions (e.g., voices resembling vivid memories of phrases heard in the past) to scenarios where voices have their private intricate opinions, preferences, motives, plans, or even memories (**Table 5**).

**Table 5:** Cluster E: voice’s own life.

ID #	Statement	Mean score
25	The voice has no life of its own, it is like an echo of the voice-hearer’s thoughts and feelings, or like a living memory of statements heard in the past.	1.1250
7	The voice can react regardless of the voice-hearer’s will: it activates and experiences different moods or emotions.	2.4583
32	The voice can react regardless of the voice-hearer’s will; it activates, experiences different moods or emotions, and has its own individual opinions on various topics.	3.4583
6	The voice can react regardless of the voice-hearer’s will: it activates, experiences different moods or emotions, has its own individual opinions on various topics and preferences (likes or dislikes certain foods, places, people or things). It also has its own aspirations or plans.	4.3333
13	The voice can react regardless of the voice-hearer’s will – it activates, experiences different moods or emotions, has its own individual opinions on various topics and preferences (likes or dislikes certain foods, places, people or things). It also has its own aspirations or plans and has its own memories to which the voice-hearer has no access.	5.0000

### Cluster F: voice influence

While auditory hallucinations themselves are often ego-dystonic experiences, disowned and perceived as alien, this dimension additionally encompasses unwanted and distressing thoughts, feelings, sensations, or perceptions induced by voices that intrude into consciousness. In the context of dissociation, this may involve intrusions of dissociated parts of the self, leading to disruptions in identity, memory, or perception, and potentially even usurping executive control. For some patients, it may be difficult to clearly distinguish between ego-syntonic reactions to voices (e.g., feeling afraid or paralyzed) and intrusions of ego-dystonic content. In such cases, it is advisable to code the lower level of complexity (**Table 6**).

**Table 6:** Cluster F: voice influence.

ID #	Statement	Mean score
24	When the voice evokes reactions, the voice-hearer experiences them as their own (egosyntonic), responding appropriately to the stimulus.	1.1739
12	The voice can induce emotions and thoughts in the voice-hearer, which are experienced egodystonically.	2.3333
28	The voice can induce emotions, thoughts, bodily sensations, or other perceptual experiences (e.g., visions) in the voice-hearer, which are experienced egodystonically.	3.2083
5	The voice can induce emotions, thoughts, bodily sensations, or other perceptual experiences (e.g., visions), and take over motor control (speech or action) over the voice-hearer, which is not covered by amnesia.	4.1667
8	The voice can induce emotions, thoughts, bodily sensations, or other perceptual experiences (e.g., visions), and take over motor control (speech or action) over the voice-hearer, which is covered by amnesia.	4.9167

### Cluster X: voice’s vocal characteristics

Two statements in this cluster described the characteristics of the voice in terms of tone, timbre, or accent. Based on these features, some voice-hearers may perceive the voice as representing a certain gender or age group if it possesses human-like characteristics (**Table 7**).

**Table 7:** Cluster X: voice’s vocal characteristics.

ID #	Statement	Mean score
19	The voice has no characteristic accent, timbre or tone. Its gender or age cannot be identified (e.g. child, adult).	1.4167
27	A voice has a distinctive accent, timbre or tone so that its age or gender can be determined.	3.0833

### Application task

In this task, we used the procedures of content analysis to code two interview transcripts referenced in the preparation step. This allowed us to evaluate the levels of voice complexity experienced by participants. Content analysis is a well-established qualitative research method used to systematically analyze textual data and derive meaningful insights (15). Prior to coding, raters were provided with the codebook developed through CM and trained how to use it. Instructions for coding are outlined in **Appendix 2**.

Inter-rater reliability was evaluated using IBM SPSS v29 by examining the coding decisions made by seven raters, (four psychiatrists and three psychologists), all with psychotherapeutic experience and working with people who hear voices. Their mean years of clinical experience was 15.43 (SD = 8.02). They all received training in scoring the SCIV and were supervised by the first author of this manuscript. The training took place in a webinar discussing the codebook and scoring procedures. Raters independently coded two SCIV transcripts. Kendall’s Coefficient for A-F categories in the first interview (W = .613) indicated substantial agreement between raters and almost perfect agreement (W = .805) in the second one (16). All judges fully agreed on category X in both interviews. Overall, these results demonstrate good agreement among raters in assessing VC dimensions (see **Table 8** for the distribution of scores).

**Table 8:** Rater assessments of voice complexity in two interviews (Int1 & Int2) with descriptive statistics.

Category	Mean	SD	Mean Rank	R1	R2	R3	R4	R5	R6	R7
Int1_A	3	0	4.86	3	3	3	3	3	3	3
Int1_B	3.14	.378	5.07	3	3	3	3	3	4	3
Int1_C	2	0	2.21	2	2	2	2	2	2	2
Int1_D	2.14	.378	2.57	2	2	2	2	2	2	3
Int1_E	2.71	.488	4.07	3	3	3	2	3	3	2
Int1_F	1.71	.951	2.21	1	1	1	3	3	2	1
Int1_X	2	0		2	2	2	2	2	2	2
Int2_A	4	0	1.21	4	4	4	4	4	4	4
Int2_B	4.57	.535	2.93	4	5	5	5	5	4	4
Int2_C	5	0	4.21	5	5	5	5	5	5	5
Int2_D	5	0	4.21	5	5	5	5	5	5	5
Int2_E	5	0	4.21	5	5	5	5	5	5	5
Int2_F	5	0	4.21	5	5	5	5	5	5	5
Int2_X	2	0		2	2	2	2	2	2	2

## Discussion

The findings of this study contribute to our understanding of auditory verbal hallucinations (AVH) by operationalizing the concept of VC through a participatory mixed-methods approach. The identification of seven dimensions of VC provides a nuanced framework for clinicians and researchers to assess and differentiate the complexity of AVH across diverse clinical populations. The development of a comprehensive codebook facilitates the systematic evaluation of VC, offering a standardized approach for clinicians to identify patients who may benefit from dissociation-based therapy approaches.

There are currently no guidelines for tailoring therapeutic interventions to different presentations of AVH. While some approaches, such as CBT for psychosis, may be suitable for a range of voices, others are primarily designed for more complex voices. An example of the latter is the clinical trial ‘Talking with Voices’ (17), which requires the voice to have the capacity to engage in dialogue, indicating a certain level of complexity. However, beyond this codebook, no comprehensive and standardized method for operationalizing voice complexity has been established.

The recognition of AVH as transdiagnostic phenomena underscores the importance of developing assessment tools capable of capturing the multifaceted nature of voices across different clinical groups. Our study addresses this gap by delineating dimensions of VC that go beyond traditional conceptualizations of AVH. By involving both healthcare professionals and voice-hearers in the conceptualization process, we ensured the incorporation of diverse perspectives, enhancing the ecological validity and clinical relevance of the developed codebook. The dimensions of VC identified in this study show their multifaceted nature, but they explore voices primarily through dissociative lenses. Previous research has suggested a continuum of psychosis-dissociation, indicating that voices may exhibit dissociative characteristics such as autonomy and personification (18, 19). Our findings support and extend these perspectives by providing a structured framework to assess the complexity of voices, including their content, interaction with the voice-hearer, and influence.

The application of the codebook to interview transcripts demonstrated substantial inter-rater reliability (16), indicating the reliability of the coding scheme across different raters. This reliability is crucial for ensuring the consistency and validity of assessments, particularly in clinical settings where treatment decisions may be based on the evaluation of VC.

Two interview transcripts that demonstrated participants’ sensitivity to the problem being investigated were used in the application task and individually tested by seven raters. Results showed not only very good inter-rater agreement among competent judges but also a significant difference in voice complexity between a patient diagnosed with schizophrenia and one with dissociative identity disorder, indicated by the general scores and particular sub-scales. This confirmed our expectations and was consistent with literature showing that voices representing dissociative parts in dissociative identity disorder can be quite complex (20).

It can be expected that the distribution of scores across different dimensions of VC will vary across different groups of voice-hearers. Studies show that those in clinical groups have less sense of control over AVH compared to non-clinical voice-hearers, which in turn affects the level of distress (21). It can be assumed that particularly two dimensions of complexity – ‘Voice Influence’ and ‘Voice’s ‘Own Life’’ – may contribute to this phenomenon. Therefore, it is important to investigate the relationships between these various dimensions of complexity and the levels of experienced distress.

One can also expect that scores will not be stable over time. Due to phobia of inner experience (5), many voice-hearers are reluctant to make contact with voices and talk about them. In time, they may become aware or disclose a greater level of complexity, including System Complexity (22). There are frequently many conflicts among the voices and between voices and the voice-hearer. These inner dynamics may change in the course of therapy, affecting dimensions such as Content Complexity, Interaction Complexity with Voice-Hearer, or Voice Influence. Overcoming the phobia of inner experiences and realizing there is amnesia for daily events can also make patients more aware of the Voice’s Own Life. Further studies comparing these dimensions of VC across groups may lead to a better understanding of auditory hallucinations and more informed treatment planning. Future developments in this area could also involve refining the codebook based on feedback from clinicians and exploring the applicability of this framework in voice-hearers representing different clinical groups.

## Conclusion

The establishment of a standardized codebook for assessing voice complexity marks a significant advance in our understanding of AVH. By delineating its dimensions and providing a systematic assessment framework, this study equips clinicians and researchers with essential tools for comprehensively evaluating AVH across diverse clinical contexts. This standardized approach has the potential to stimulate comparative studies across varied clinical samples and inform therapeutic decisions tailored to the unique nature of patients’ voice experiences.

## Data Availability

The original contributions presented in the study are included in the article/**Supplementary Material**. Further inquiries can be directed to the corresponding author.

## References

[1] Waters F Blom J Jardri R Hugdahl K Sommer I. Auditory hallucinations, not necessarily a hallmark of psychotic disorder. psychol Med. (2018) 48:529–36. doi: 10.1017/S0033291717002203 28826411

[2] Slotema CW Daalman K Blom JD Diederen KM Hoek HW Sommer IEC. Auditory verbal hallucinations in patients with borderline personality disorder are similar to those in schizophrenia. psychol Med. (2012) 42:1873–8. doi: 10.1017/S0033291712000165 22336487

[3] Daalman K Diederen KM. A final common pathway to hearing voices: examining differences and similarities in clinical and non-clinical individuals. Psychosis. (2013) 5:236–46. doi: 10.1080/17522439.2013.796402

[4] Longden E Branitsky A Moskowitz A Berry K Bucci S Varese F. The relationship between dissociation and symptoms of psychosis: a meta-analysis. Schizophr Bull. (2020) 46:1104–13. doi: 10.1093/schbul/sbaa037 PMC7505175 32251520

[5] Van der Hart O Nijenhuis ER Steele K. The haunted self: Structural dissociation and the treatment of chronic traumatization. New York/London: WW Norton & Company (2006).

[6] Woods A Jones N Alderson-Day B Callard F Fernyhough C. Experiences of hearing voices: analysis of a novel phenomenological survey. Lancet Psychiatry. (2015) 2:323–31. doi: 10.1016/S2215-0366(15)00006-1 PMC4580735 26360085

[7] Alderson-Day B Woods A Moseley P Common S Deamer F Dodgson G. Voice-hearing and personification: characterizing social qualities of auditory verbal hallucinations in early psychosis. Schizophr Bull. (2021) 47:228–36. doi: 10.1093/schbul/sbaa095 PMC7824995 33484268

[8] Holmes EA Brown RJ Mansell W Fearon RP Hunter EC Frasquilho F. Are there two qualitatively distinct forms of dissociation? A review and some clinical implications. Clin Psychol Rev. (2005) 25:1–23. doi: 10.1016/j.cpr.2004.08.006 15596078

[9] Laddis A Dell PF. Dissociation and psychosis in dissociative identity disorder and schizophrenia. J Trauma Dissociation. (2012) 13:397–413. doi: 10.1080/15299732.2012.664967 22651674

[10] Kane M Trochim WM. Concept mapping for planning and evaluation. Thousand Oaks, CA: Sage Publications, Inc (2007).

[11] Pietkiewicz IJ Tomalski R Gonzalez A Eyles T. Structured Clinical Interview for Voice-Hearers (version 1.5) [Unpublished manuscript]. ResearchGate (2019). doi: 10.13140/RG.2.2.27013.09446/1

[12] Kruskal JB Wish M. Multidimensional scaling. London: Sage (1978).

[13] Davison ML. Multidimensional scaling. New York: John Wiley & Sons (1983).

[14] Ward JJH. Hierarchical grouping to optimize an objective function. J Am Stat Assoc. (1963) 58:236–44. doi: 10.1080/01621459.1963.10500845

[15] Krippendorff K. Content analysis: An introduction to its methodology (4th ed.). Thousand Oaks, CA: Sage Publications (2018).

[16] Landis JR Koch GG. The measurement of observer agreement for categorical data. Biometrics. (1977) 33:159–74. doi: 10.2307/2529310 843571

[17] Longden E Corstens D Bowe S Pyle M Emsley R Peters S. A psychological intervention for engaging dialogically with auditory hallucinations (Talking With Voices): A single-site, randomised controlled feasibility trial. Schizophr Res. (2022) 250:172–9. doi: 10.1016/j.schres.2022.11.007 PMC9754007 36423442

[18] Moskowitz A Mosquera D Longden E. Auditory verbal hallucinations and the differential diagnosis of schizophrenia and dissociative disorders: Historical, empirical and clinical perspectives. Eur J Trauma Dissociation. (2017) 1:37–46. doi: 10.1016/j.ejtd.2017.01.003

[19] Ross CA. Voices: are they dissociative or psychotic? J Nervous Ment Dis. (2020) 208:658–62. doi: 10.1097/NMD.0000000000001206 32868688

[20] Dorahy MJ Shannon C Seagar L Corr M Stewart K Hanna D. Auditory hallucinations in dissociative identity disorder and schizophrenia with and without a childhood trauma history: Similarities and differences. J nervous Ment Dis. (2009) 197:892–8. doi: 10.1097/NMD.0b013e3181c299ea 20010024

[21] de Leede-Smith S Barkus E. A comprehensive review of auditory verbal hallucinations: lifetime prevalence, correlates and mechanisms in healthy and clinical individuals. Front Hum Neurosci. (2013) 7:367. doi: 10.3389/fnhum.2013.00367 23882203 PMC3712258

[22] Boon S Steele K Van Der Hart O. Coping with trauma-related dissociation: Skills training for patients and therapists. New York/London: WW Norton & Company (2011).

