# Supplementary material for: Developing a codebook for assessing auditory hallucination complexity using mixed methods
Source: Front Psychiatry. 2024 Dec 12;15:1441919. doi: 10.3389/fpsyt.2024.1441919 (PMC11669670; doi:10.3389/fpsyt.2024.1441919)
Supplement: Supplementary file 1 [file Supplementaryfile1.pdf]

Appendix 1: Voice Complexity Codebook

A: SYSTEM COMPLEXITY	B: CONTENT COMPLEXITY	C: VOICE'S INTEREST COMPLEXITY	D: INTERACTION COMPLEXITY WITH VOICE-HEARER	E: VOICE'S 'OWN LIFE'	F: VOICE INFLUENCE	X: VOICE VOCAL CHARACTERISTICS
**A1**: A person experiences one voice.	**B1**: No verbal content, simple sounds (e.g. noise, rustling, squealing, crying, screaming, knocking, music).	**C1**: It is not possible to determine voices' object of interest.	**D1**: The voice does not respond to the voice-hearer (e.g. his or her attempts to establish contact) or no such attempts have been made.	**E1**: The voice has no life of its own, it is like an echo of the voice-hearer's thoughts and feelings, or like a living memory of statements heard in the past.	**F1**: When the voice evokes reactions, the voice-hearer experiences them as their own (egosyntonic), responding appropriately to the stimulus.	**X1**: The voice has no characteristic accent, timbre or tone. Its gender or age cannot be identified (e.g. child, adult).
**A2**: One experiences several different voices which do not interact or know about each other's existence.	**B2**: Verbal content is difficult to identify, containing incomprehensible statements (e.g. whispers, chatter).	**C2**: The voice focuses on the voice-hearer and regulating his or her behavior or emotions.	**D2**: The voice understands the questions or content addressed to it and responds in a simple way (e.g., gives casual answers, makes faces or gestures, or changes behavior).	**E2**: The voice can react regardless of the voice-hearer's will: it activates, experiences different moods or emotions.	**F2**: The voice can induce emotions and thoughts in the voice-hearer, which are experienced egodystonically.	**X2**: A voice has a distinctive accent, timbre or tone so that its age or gender can be determined.
**A3**: One experiences several different voices with simple and repetitive patterns of interaction (e.g., attacking each other).	**B3**: Poor content limited to simple words or short, repetitive phrases.	**C3**: The voice focuses on the voice-hearer, other people, or situations.	**D3**: The voice understands the questions or content addressed to it and one can start a simple conversation with it (a sequence of several two-way statements).	**E3**: The voice can react regardless of the voice-hearer's will; it activates, experiences different moods or emotions, has its own individual opinions on various topics.	**F3**: The voice can induce emotions, thoughts, bodily sensations, or other perceptual experiences (e.g., visions) in the voice-hearer, which are experienced egodystonically.	
**A4**: One experiences several different voices, with complex interactions, and their patterns vary depending on the situation (e.g., sometimes they attack each other and other times they are in agreement).	**B4**: The content of voices includes complex statements, comments, or opinions on limited topics.	**C4**: The voice focuses on the voice-hearer, other people or situations, and its own emotional states, needs, or plans.	**D4**: The voice understands the questions or content addressed to it and it is possible to maintain a fluent conversation with it for a longer period.	**E4**: The voice can react regardless of the voice-hearer's will: it activates, experiences different moods or emotions, has its own individual opinions on various topics and preferences (likes or dislikes certain foods, places, people or things). It also has its own aspirations or plans.	**F4**: The voice can induce emotions, thoughts, bodily sensations, or other perceptual experiences (e.g., visions), and take over motor control (speech or action) over the voice-hearer, which is not covered by amnesia.	
**A5**: There are several or even a dozen voices which can be organized into different groups or subsystems. There are complex interactions between them which change over time.	**B5**: The content of voices includes complex statements, comments, or opinions on various topics that change dynamically.	**C5**: The voice focuses on the voice-hearer, other people, or situations, its own emotional states, needs or plans, and the experiences of other voices.	**D5**: The voice understands the questions or content addressed to it and it is possible to maintain a fluent conversation with it for an extended period, and physical contact is possible (e.g. touching, stroking, hugging, hitting the voice).	**E5**: The voice can react regardless of the voice-hearer's will – it activates, experiences different moods or emotions, has its own individual opinions on various topics and preferences (likes or dislikes certain foods, places, people or things). It also has its own aspirations or plans and has its own memories to which the voice-hearer has no access.	**F5**: The voice can induce emotions, thoughts, bodily sensations, or other perceptual experiences (e.g., visions), and take over motor control (speech or action) over the voice-hearer, which is covered by amnesia.	
