# Supplementary material for: Developing a codebook for assessing auditory hallucination complexity using mixed methods
Source: Front Psychiatry. 2024 Dec 12;15:1441919. doi: 10.3389/fpsyt.2024.1441919 (PMC11669670; doi:10.3389/fpsyt.2024.1441919)
Supplement: Supplementary file 2 [file Supplementaryfile2.pdf]

Appendix 2: *Instructions for Raters*

The aim of this content analysis is to systematically review the interview transcript to assess the level of voice complexity experienced by participants. Before commencing coding, thoroughly review the codebook provided (Appendix 1). It contains predefined categories A to F, each with five options describing different levels of voice complexity. Additionally, familiarize yourself with category X, which offers only two options.

When coding a specific fragment of information from the interview transcript, focus solely on the content within that chunk. Avoid integrating information from other parts of the transcript, even if it suggests higher voice complexity. While using the predefined categories from the codebook, remain open to the possibility of identifying new codes or themes. Allow for flexibility in coding to capture subtleties and variations in voice complexity.

Coding Procedure:

- Read each fragment of information carefully, considering the nuances of language and context.
- Select the most appropriate option from categories A to F or category X that best reflects the level of voice complexity expressed in that fragment, e.g.: *Actually, I've never heard any other voices except this one, that is so unpleasant*. Code A1 is appropriate for this chunk relating to the complexity of the system.
- Note that a single fragment of information may encompass different aspects of voice complexity, allowing for the use of more than one code for the same fragment.

Ensure consistency and accuracy in coding by closely adhering to the definitions and criteria outlined for each category in the codebook. If necessary, refer back to the codebook to clarify coding decisions.

After coding the entire interview, specify the highest level of voice complexity in each of the seven categories.
